# Maternal hemoglobin change from early pregnancy to second trimester is associated with risk of gestational diabetes mellitus: a retrospective cohort study

**DOI:** 10.3389/fnut.2023.1197485

**Published:** 2023-06-15

**Authors:** Husni Zain Sulhariza, Mohd Shariff Zalilah, Appannah Geeta

**Affiliations:** ^1^Department of Nutrition, Faculty of Medicine and Health Sciences, Universiti Putra Malaysia, Serdang, Selangor, Malaysia; ^2^Institute of Public Health, National Institute of Health, Ministry of Health Malaysia, Shah Alam, Selangor, Malaysia

**Keywords:** gestational diabetes mellitus, hemoglobin level, hemoglobin change, iron supplement, retrospective cohort

## Abstract

**Introduction:**

The accrual of iron that is reflected in high maternal hemoglobin (Hb) status is increasingly recognized as a risk factor for gestational diabetes mellitus (GDM). Changes in maternal Hb level could also implicate glycemic status in pregnancy. This study aimed to determine the associations between maternal Hb levels and their changes with GDM.

**Methods:**

In this retrospective cohort study, a total of 1,315 antenatal records of mothers with singleton pregnancies from eight health clinics of a district in the northern region of Peninsular Malaysia who delivered between 1st January 2016–31st December 2017 were analyzed. Data extracted from the records were socio-demographic, anthropometric, obstetrical, and clinical data. Hb levels were extracted at booking (<14 weeks) and second trimester (14–28 weeks). Change in Hb was determined by subtracting the Hb level in the second trimester from the booking Hb level and was categorized as decreased, unchanged, and increased Hb. The associations between maternal Hb levels and their changes with GDM risk were analyzed using multiple regression, adjusting for covariates in four different models. Model 1: maternal age and height. Model 2: covariates of Model 1 added with parity, history of GDM, and family history of diabetes. Model 3: covariates of Model 2 added with iron supplementation at booking. Model 4: covariates of Model 3 added with Hb level at booking.

**Results and Discussions:**

Unchanged Hb level from booking to second trimester was significantly associated with GDM risk in Model 1 (AOR: 2.55; 95% CI: 1.20, 5.44; *p* < 0.05), Model 2 (AOR: 2.45, 95% CI: 1.13, 5.34; *p* < 0.05) Model 3 (AOR: 2.42; 95% CI: 1.11, 5.27; *p* < 0.05), and Model 4 (AOR: 2.51; 95% CI: 1.15, 5.49; p < 0.05). No significant associations were observed between maternal Hb levels and GDM in the study.

**Conclusion:**

Unchanged Hb levels from the booking (<14 weeks of gestation) to the second trimester (14–28 weeks) increased GDM risk. Further investigation is warranted to evaluate the associations between changes in maternal Hb and GDM risk and to identify potential factors influencing this relationship.

## Introduction

Hyperglycemia that is first detected at any time during pregnancy can be classified as either diabetes in pregnancy (DIP) or gestational diabetes mellitus (GDM) (1). DIP may be an undiagnosed type 2 diabetes mellitus (T2DM) or “overt diabetes” identified in the first trimester while GDM develops in the second and third trimesters (2–5). Globally, an estimated 21.1 million live births were exposed to some forms of hyperglycemia in pregnancy, of which 80.3% were due to GDM (6). GDM prevalence is generally considered to be somewhere from 1 to 28% of all pregnancies. The variation in prevalence rates could also be related to the diversity of the populations studied, the screening methods, and the diagnostic criteria used. Nevertheless, with the increasing prevalence of T2DM, the incidence of GDM is also on the rise wherever the rate of obesity is prevalent (7–9). In the current global epidemic of diabetes, the age of onset has decreased significantly, which concurrently affects a significant proportion of reproductive-age women (7–9).

In 2016, the national surveillance on Maternal and Child Health reported that the prevalence of hyperglycemia during pregnancy in Malaysia was 13.5% (10). However, this report did not distinguish the types of maternal hyperglycemia. A systematic review reported a pooled prevalence of GDM in Malaysia at 21.5% (11). The high prevalence was due to the high heterogeneity of studies that could be influenced by subject recruitment, diagnosis criteria, screening standards, and the definition of GDM (11). On the contrary, two systematic reviews of the GDM pooled prevalence in Asian countries reported a lower prevalence (10.1–11.5%) in Malaysia, in which only two local studies were included in the analysis (12, 13).

Iron accumulation is increasingly recognized to be associated with an increased risk of T1DM and has been proposed to be involved in the pathophysiological mechanism of T2DM (14) as well as GDM (15, 16). Although iron is essential for many important metabolic processes, the excessive iron level may be pathological. Through the Fenton reaction, iron can generate reactive oxygen leading to oxidative stress, affecting the β-cells to dysfunction and may reduce insulin secretion and increases insulin resistance (17). Hemoglobin (Hb) is the largest body iron pool in the body that is commonly used as a crude iron biomarker (18). Maternal anemia and high Hb concentration during pregnancy have been reported to increase the risks of adverse pregnancy outcomes and maternal health (19–22). Pregnant women with Hb <11.0 g/dL are considered anemic and Hb levels within the normal range (11.0–14.0 g/dL) during pregnancy are considered beneficial for the well-being of the mother and fetus (23). Anemia in pregnancy is associated with poor maternal outcomes such as preterm birth, low birth weight, infection, and neonatal complications (21, 24). On the other hand, high maternal Hb increases blood viscosity, causing placental perfusion that leads to poor maternal-fetal exchange which would result in adverse pregnancy outcomes such as low birth weight, preterm births, preeclampsia, GDM and stillbirths (25–27).

There is increasing evidence of the relationship between elevated Hb and GDM (25, 28, 29). However, the available literature reported mixed findings compared to the association of Hb status with T1DM and T2DM. During pregnancy, women need to have Hb level ≥ 11.0g/dL to ensure an uncomplicated pregnancy, healthy development of the fetus, and maturity of the newborn child. In Malaysia, efforts to ensure a lower incidence of iron deficiency anemia (IDA) in pregnancy include the practice of iron supplementation as prophylaxis or even as a treatment and dietary advice on the intake of iron-rich foods (30, 31). High maternal Hb level has always been perceived as a sign of good nutritional status rather than as a contributing factor to adverse pregnancy outcomes, such as GDM. It has also been reported that the changes in maternal Hb levels from early to mid- or late pregnancy could implicate pregnancy outcomes (22, 32), but none are specific to GDM. As there are gaps in the available literature on the relationships between maternal Hb level and its changes with GDM, this study aimed to determine the associations of Hb concentrations at booking and second trimester and changes in Hb concentrations from booking (<14 weeks) to the second trimester (14–28 weeks) with GDM risk.

## Methods

### Data sources

Data for this retrospective cohort study was obtained from antenatal cards in eight health clinics (HCs) in Kuala Muda, Kedah. The antenatal card is a well-established record by the Ministry of Health Malaysia (MOH). Women receiving antenatal care services in HCs are issued two copies of antenatal cards, one kept by the women and the second by HCs. For this study, data were derived from the second copy of antenatal cards of women who delivered from January 2016 to December 2017. Maternal information extracted from the antenatal cards were socio-demographic characteristics (e.g., maternal age, educational level, ethnicity, and follow-up date), obstetrical information (e.g., gravidity, parity, history of GDM, and family history of diabetes), anthropometric measurements (e.g., weight at booking and height), and clinical information (e.g., maternal glucose, Hb, and iron supplementation).

### Sample size and study population

Using a formula for cohort study (33), the minimum sample needed for this study was 430 women, based on the prevalence of GDM in Malaysian women (11.8%) (34). The inclusion criteria were Malaysian, singleton pregnancy with a complete antenatal record from booking until the second oral glucose tolerance test (OGTT) in the second trimester. The exclusion criteria included maternal age < 19 years old, incomplete OGTT records, diabetes, blood disorders, cardiovascular disease, infectious disease, thyroid disorder, and placenta previa.

**Figure 1:**
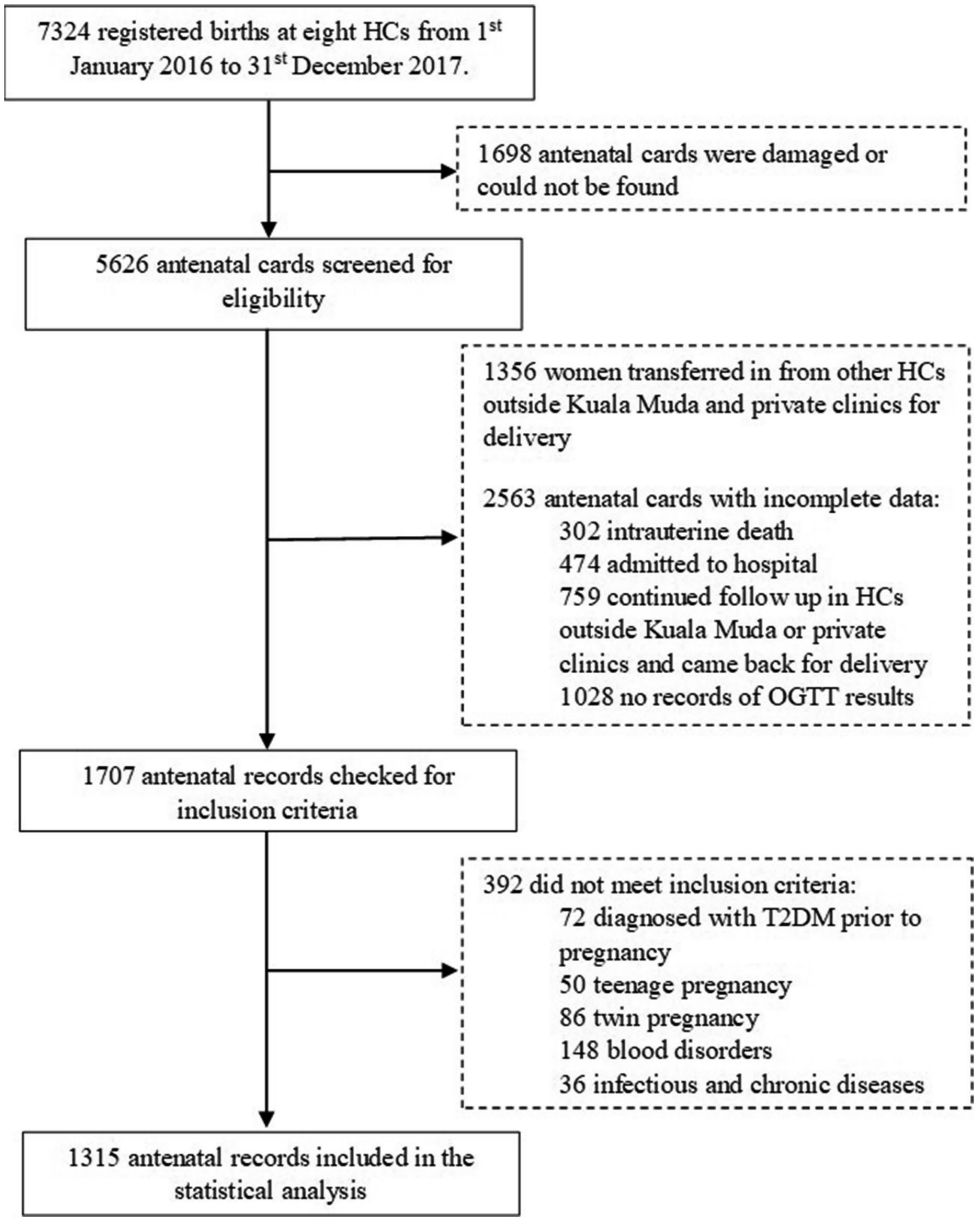
Selection process of the antenatal records.

There were 7,324 registered births for the eight HCs from 1st January 2016 to 31st December 2017. Of the total number, 1698 antenatal cards were damaged or could not be found, leaving 5,626 cards available for screening. About 1,356 women had antenatal care elsewhere and attended antenatal visits in Kuala Muda after 32 weeks. A total of 2,563 antenatal cards had incomplete records of biochemical and clinical data between booking until the second trimester with 302 women had miscarriage or intrauterine death (IUD), 402 were admitted to the hospital before the second OGTT due to various medical conditions, 759 women made the antenatal booking at the HCs but continued to follow up in other antenatal care clinics but came back for delivery, and 1,028 with no records of OGTT. The remaining 1707 were checked for inclusion and exclusion criteria. The final sample included in this study comprised 1,315 antenatal records with complete data on two times OGTT, biochemical, and clinical data (Figure 1).

### Maternal data and other measurements

The socio-demographic information included the date of birth, ethnicity, and education level. Age of women was calculated by subtracting the date of birth from the date of the first antenatal visit (booking). Obstetrical information extracted were gravidity, parity, history of GDM, and family history of diabetes. As pre-pregnancy weight was not available in the study, weight at booking (4th–13th week of pregnancy) was used to estimate the pre-pregnancy body mass index (BMI). BMI was calculated and categorized according to the World Health Organization classification; underweight (<18.5 kg/m^2^), normal weight (18.5–24.9 kg/m^2^), overweight (25.0–29.9 kg/m^2^), and obese (≥30.0 kg/m^2^) (35).

Women in this study underwent a 1-step 75g OGTT before 14 weeks of gestation (OGTT 1) and was repeated during 24–34 weeks of gestation (OGTT 2). The screening involved a fasting glucose plasma (FPG) test and a 2-h postprandial (2-HPP). GDM was diagnosed if the results were ≥ 5.6 mmoL/L for FPG and ≥ 7.8 mmoL/L for 2-HPP (MOH, 2013). Hb levels were extracted at booking (4th–13th week) and at the highest gestational week of each trimester that was available (first trimester: 4th–13th week; second trimester: 14th–28th week). The study used a reference of Hb concentration based on the low Hb cut-offs of <11.0 g/dL, also classified as anemia in pregnancy (30, 36) and used a suggestion of Hb ≥13.0 g/dL from a meta-analysis study as high Hb reference (25). Change in Hb from booking until the second trimester was calculated to measure the difference of Hb value at booking compared to Hb value at the second trimester. The change in Hb was calculated by subtracting the Hb value at the second trimester from the Hb value at booking (second trimester Hb–booking Hb). The Hb change was then categorized as decreased, unchanged, and increased Hb value in the second trimester.

### Iron supplementation

Information on iron supplements consumed included the type of iron supplement, dosage, and duration of intake from booking until the second trimester, whether prescribed by the health personnel or self-purchased by the women. The reported types of iron supplements in the study were ferrous fumarate, ferrous sulfate, ferrous gluconate, iron (III)-hydroxide polymaltose complex (IPC), and iron dextran. The dosage and duration of iron supplement intake were calculated based on the following formula:

Duration of iron supplement intake (week):

Gestationalweekofthesecondtrimester−gestationalweekofbooking

The daily dosage of iron supplement intake:

$$\frac{Totaldosageofironsupplementintake\;\left(bookinguntilthesecondtrimester\right)}{\left(Durationofironsupplementintake\right)\times \left(7\;days\right)}$$

The daily dosage of iron supplement intake was classified based on the iron supplement daily intake recommendation for pregnancy (37). The recommendation for daily oral iron supplementation to prevent maternal anemia is 30 mg to 60 mg of elemental iron. For a pregnant woman with anemia (Hb <11.0 g/dL), the elemental iron should be increased to 120 mg daily until the Hb level increases to ≥11.0 g/dL (37).

### Statistical analysis

All continuous data were tested for normality and presented as mean and standard deviation (SD), while the median was reported for variables that did not comply with normal distribution. Descriptive statistics (mean, standard deviation, frequency, and percentage) were used to describe the data. Comparisons of continuous variables between groups were examined using the *t*-test and analysis of variance (ANOVA) wherever applicable. The chi-square (*χ*^2^) test was used for categorical variables to check for associations between variables. All covariates were tested and those with value of *p* <0.25 were considered in the analysis of maternal Hb status and GDM risk relationship. Four models were built in the multivariate logistic regression analysis. Model 1 adjusted for maternal age and height. Parity, history of GDM, and family history of diabetes were added to covariates of Model 1 as Model 2. For Model 3, iron supplementation at booking was added to the covariates of Model 2. Lastly, the Hb level at booking was added to covariates of Model 3 as Model 4. Additionally, Hb <11.0 g/dL and decreased Hb were defined as the reference group. All statistical tests were two-tailed, and the significance level was set at *p* < 0.05.

## Results

### Sample characteristics

Table 1 compares the characteristics of non-GDM and GDM women. The prevalence of GDM was 17.8% (1,081/234). GDM women (31.50 ± 5.00 years) were significantly older than non-GDM (30.20 ± 5.01 years). GDM women had a higher percentage of multiparous (24.4% vs. 18.5%), family history of diabetes mellitus (51.3% vs. 45.4%), and more history of GDM (17.1% vs. 5.4%) than non-GDM women. There was a significant difference in height between non-GDM (1.56 ± 0.06 m) and GDM (1.55 ± 0.06 m) in which, the proportion of height < 1.50 m was higher in the GDM group (15.8% vs. 10.9%). No significant difference in booking BMI status was observed between GDM (26.51 ± 4.77 kg/m^2^) and non-GDM (26.61 ± 5.67 kg/m^2^) women. Non-GDM women (48.67 ± 28.08 mg) took a higher dose of elemental iron from iron supplements dailycompared to GDM women (46.25 ± 25.77 mg) but the difference was not significant. In this study, iron supplementation was not associated with GDM (*χ^2^* = 1.52; *p* > 0.05).

**Table 1:** Characteristics of women (*N* = 1,315).

Variables	Maternal glycemia^a^			
	Non-GDM (*n* = 1,081)	GDM (*n* = 234)	*t*-value/*χ*^2^	Value of *p*
Age in years (Mean ± SD)	30.20 ± 5.01	31.50 ± 5.00	−3.61	0.00**
<25	159 (14.7)	19 (8.2)	12.59	0.01*
25–29	334 (30.9)	64 (27.4)		
30–34	348 (32.2)	80 (34.2)		
≥35	240 (22.2)	71 (30.2)		
Education level in years (Mean ± SD)	13.16 ± 2.80	13.12 ± 2.81	0.20	0.84
Secondary and lower	568 (52.6)	126 (53.9)	0.30	0.96
STPM/diploma/certificate	291 (26.9)	59 (25.2)		
Higher institution	222 (20.5)	49 (20.9)		
Occupation status				
Working	666 (61.6)	143 (61.1)	0.02	0.89
Not working	415 (38.4)	91 (38.9)		
Parity (Mean ± SD)	1.37 ± 1.27	1.55 ± 1.43	−1.82	0.07
0	343 (31.7)	68 (29.0)	4.53	0.21
1	285 (26.4)	61 (26.1)		
2	253 (23.4)	48 (20.5)		
≥3	200 (18.5)	57 (24.4)		
Medical history of GDM	58 (5.4)	40 (17.1)	38.37	<0.00**
Family history of diabetes	491 (45.4)	120 (51.3)	2.66	0.10
Height in meters (Mean ± SD)	1.56 ± 0.06	1.55 ± 0.06	2.45	0.01*
<1.50	118 (10.9)	37 (15.8)	5.27	0.15
1.50–1.55	368 (34.0)	73 (31.2)		
1.56–1.60	349 (32.3)	78 (33.3)		
>1.60	246 (22.8)	46 (19.7)		
Booking^c^ BMI (kg/m^2^) (Mean ± SD)	26.61 ± 5.67	26.51 ± 4.77	0.27	0.79
Underweight (<18.5)	72 (6.7)	9 (3.8)	5.49	0.14
Normal (18.5–24.9)	378 (35.0)	88 (37.6)		
Overweight (25.0–29.9)	353 (32.7)	87 (37.2)		
Obese (≥30.0)	278 (25.6)	50 (21.4)		
Iron supplementation at booking			4.68	0.10
Yes	660 (61.1)	156 (66.6)		
No	421 (38.9)	78 (33.4)		
Elemental iron intake daily from booking^b^ to second trimester (mg)	48.67 ± 28.08	46.25 ± 25.77	1.21	0.23
Duration of intake (week)	19.42 ± 3.49	19.69 ± 3.45	−1.07	0.29
Daily dosage (mg)^c^				
≤60.00	788 (72.9)	179 (76.8)	1.52	0.22
>60.00	293 (27.1)	54 (23.2)		

^a^GDM was classified according to the Ministry of Health Malaysia (30) criteria as either or both FPG ≥ 5.6 mmoL/L or 2-h postprandial ≥ 7.8 mmoL/L.

^b^First antenatal visit at < 14^th^ week of gestational age.

^c^Classification of daily elemental iron supplement dosage was based on WHO (36) recommendation.

*p < 0.05; **p < 0.01.

### Hemoglobin status

No significant difference was observed in Hb concentration at booking and the second trimester between non-GDM and GDM women (Table 2). GDM women were more likely to have Hb concentration ≥ 13.0 g/dL (booking = 38.5% vs. 34.7%; second trimester = 11.2% vs. 10.0%) compared to non-GDM. The change of Hb from booking to the second trimester was significantly associated with maternal glycemia status (*χ^2^* = 6.29; *p* < 0.05) but no significant mean difference was observed between GDM and non-GDM groups (*t* = −0.27; *p* > 0.05). More non-GDM women had decreased Hb than GDM women (82.1% vs. 80.8%), while the proportion of unchanged Hb levels was higher among GDM compared to non-GDM (4.7% vs. 1.9%). Table 3 presents Hb concentration level stratified by change of Hb from booking to the second trimester (decreased, unchanged and increased). The increased group had significantly lower Hb concentration at booking than the decreased and unchanged group (*F* = 122.20; *p* < 0.01). In the second trimester, the Hb concentration of the decreased group was significantly lower than the unchanged and the increased groups (*F* = 103.67; *p* < 0.01). It was also observed that Hb status at booking and second trimester were significantly associated with Hb changes (*p* < 0.01). Additionally, the GDM incidence was significantly dependent on the Hb changes, whereby the unchanged Hb had the highest proportion of GDM incidence (34.4%) than the other groups (*χ^2^* = 6.29; *p* < 0.05).

**Table 2:** Hemoglobin status of women (*N* = 1,315).

	Maternal glycemia^a^			
	Non-GDM (*n* = 1,081)	GDM (*n* = 234)	*t*-value/*χ*^2^	Value of *p*
Hemoglobin level at booking ^b^ (g/dL) (Mean ± SD)	12.63 ± 1.13	12.70 ± 1.16	−9.84	0.40
<11.0	68 (6.3)	11 (4.7)	1.74	0.42
11.0–12.9	638 (59.0)	133 (56.8)		
≥13.0	375 (34.7)	90 (38.5)		
Hemoglobin level of second trimester (g/dL) (Mean ± SD)	11.62 ± 1.06	11.71 ± 0.95	−1.22	0.22
<11.0	230 (21.3)	38 (16.2)	3.06	0.22
11.0–12.9	743 (68.7)	170 (72.6)		
≥13.0	108 (10.0)	26 (11.2)		
Change of hemoglobin: second trimester–booking (Mean ± SD)	−1.01 ± 1.20	−0.99 ± 1.13	−0.27	0.79
Decreased^c^ (−1.39 ± 0.88)	888 (82.1)	189 (80.8)	6.29	0.04*
Unchanged^d^ (0)	21 (1.9)	11 (4.7)		
Increased^e^ (0.84 ± 0.79)	172 (15.9)	34 (14.5)		

^a^GDM is classified according to the Ministry of Health Malaysia (30) criteria as either or both FPG ≥ 5.6 mmoL/L or 2-h postprandial ≥ 7.8 mmoL/L.

^b^First antenatal visit at < 14^th^ week of gestational age. ^c^Hemoglobin values of second trimester < booking.

^d^Hemoglobin values of second trimester = booking.

^e^Hemoglobin values of second trimester > booking.

*p < 0.05; **p < 0.01.

**Table 3:** Hemoglobin level and GDM incidence stratified by hemoglobin changes^a^ (*N* = 1,315).

Hemoglobin level (g/dL)	Decreased^b^ Hb (*n* = 1,077)	Unchanged^c^ Hb (*n* = 32)	Increased^d^ Hb (*n* = 206)	*F*-value /*χ*^2^	Value of *p*
Booking^e^ (Mean ± SD)	12.84 ± 1.03 ^x^	12.51 ± 1.10 ^y^	11.60 ± 1.09 ^x,y^	122.20	<0.01**
(Range)	(9.50–16.60)	(10.60–15.40)	(8.30–14.30)		
<11.0	33 (3.1)	3 (9.4)	43 (16.5)	164.56	<0.01**
11.0–12.9	557 (51.7)	19 (59.4)	146 (70.9)		
≥13.0	487 (45.2)	10 (31.2)	17 (8.3)		
Gestational week of measurement	9.23 ± 2.17	8.69 ± 2.42	9.33 ± 2.26		
Second trimester (Mean ± SD)	11.46 ± 0.96 ^x,y^	12.51 ± 1.10 ^x^	12.45 ± 1.00 ^y^	103.67	<0.01**
(Range)	(7.50–14.30)	(10.60–15.40)	(8.60–15.40)		
<11.0	253 (23.5)	3 (9.4)	12 (5.8)	125.99	<0.01**
11.0–12.9	758 (70.4)	19 (59.4)	136 (66.0)		
≥13.0	66 (6.1)	10 (31.2)	58 (28.2)		
Gestational week of measurement	26.37 ± 1.73	26.47 ± 1.19	26.30 ± 1.69		
GDM, *n* (%)	189 (17.5)	11 (34.4)	34 (16.5)	6.29	0.043*

Values are presented as mean ± SD for continuous variables and *n* (%) for dichotomous variables.

^a^Hb changes were calculated by subtracting Hb at booking from the second trimester.

^b^Hb values of the second trimester < booking.

^c^Hb values of the second trimester = booking.

^d^Hb values of the second trimester > booking.

^e^First antenatal visit at < 14^th^ week of gestational age.

^x,y^Similar alphabets indicate a significant difference between groups.

* < 0.05, **p < 0.01.

### Hemoglobin status and GDM risk

Four models adjusted for various maternal covariates to investigate the associations of Hb status with the risk of GDM are presented in Table 4. Hb concentration at booking and in the second trimester was not significantly associated with GDM risk. However, women with unchanged Hb were significantly associated with the GDM risk compared to women with decreased Hb. The risk of developing GDM for women with unchanged Hb in the second trimester compared to booking was similar among the four models (2.4 to 2.6 times, *p* = 0.02).

**Table 4:** Adjusted odd ratios for the association between maternal hemoglobin and gestational diabetes mellitus.

	Model 1		Model 2		Model 3		Model 4	
	Adjusted OR	Value of *p*	Adjusted OR	Value of *p*	Adjusted OR	Value of *p*	Adjusted OR	Value of *p*
Hb level at B (g/dL)								
<11.0	1.00		1.00		1.00		-	-
11.0–12.9	1.37 [0.70, 2.67]	0.36	1.25 [0.63, 2.46]	0.52	1.30 [0.66, 2.58]	0.45	-	-
≥13.0	1.46 [0.74, 2.89]	0.27	1.39 [0.70, 2.77]	0.35	1.49 [0.74, 2.99]	0.26	-	-
Hb level at T2 (g/dL)								
<11.0	1.00		1.00		1.00		1.00	
11.0–12.9	1.39 [0.95, 2.04]	0.09	1.45 [0.98, 2.15]	0.06	1.48 [1.00, 2.19]	0.05	1.42 [0.95, 2.14]	0.09
≥13.0	1.51 [0.87, 2.63]	0.15	1.62[0.92, 2.86]	0.09	1.64 [0.93, 2.89]	0.09	1.57 [0.87, 2.84]	0.14
Change of Hb: T2 - B								
Decreased ^d^	1.00		1.00		1.00		1.00	
Unchanged ^e^	2.55 [1.20, 5.44]	0.02*	2.45 [1.13, 5.34]	0.02*	2.42 [1.11, 5.27]	0.03*	2.51 [1.15. 5.49]	0.02*
Increased ^f^	0.94 [0.63, 1.41]	0.78	0.93 [0.62, 1.40]	0.73	0.91 [0.60, 1.37]	0.65	1.00 [0.65, 1.55]	0.99

T2: second trimester. B: booking.

^d^Hb values of T2 = B.

^e^Hb values of T2 < B.

^f^Hb values of T2 > B.

Model 1–Adjusted for age and height.

Model 2–Adjusted for age, height, parity, history of GDM, and family history of diabetes.

Model 3–Adjusted for age, height, parity, history of GDM, family history of diabetes, and iron supplement intake at booking.

Model 4–Adjusted for age, height, parity, history of GDM, family history of diabetes, iron supplementation at booking, and Hb status at booking.

*p<0.05.

## Discussion

The prevalence of GDM in this study was higher than the reported prevalence in Europe, North America, and the Caribbean, whereby the median estimate of GDM prevalence was 5.8 and 7.0%, respectively (7). As the Asian population has a higher risk of GDM, a higher prevalence in the present study than in the Western population is expected. However, the GDM prevalence in the present study was also higher than in other Asian countries such as Japan (2.8%), India (8.8%), South Korea (10.5%), China (12.6%), and Iran (14.9%). The prevalence was similar to Thailand (17.1%) and Singapore (17.6%), but lower than Hong Kong (32.5%), Taiwan (38.6%) (13), and pooled prevalence in Malaysia at 21.5% (11).

High maternal Hb is associated with an increased risk of poor maternal health, namely preeclampsia and GDM (25, 28). Studies have reported different values of high Hb status in the first trimester to be associated with risk of GDM with Turkish women at ≥12.2 g/dL (38), Finnish women at >12.0 g/dL (39), and Republic of Kosovo women at ≥13.0 g /dL (40). Similarly, studies in the Middle Eastern and Asian countries (Iran, Pakistan, Palestine and Thailand) also reported a significant association between high Hb status during the first trimester with GDM but with a more consistent Hb cut-off value of ≥12.5 g/dL (41–46). In China, several studies reported a significant association between Hb concentration of >13.0 g/dL during the first trimester and increased risk of GDM (47, 48). On the contrary, the present study did not find a significant association between GDM and Hb concentration in the first trimester. The difference in mean Hb level and the proportion of elevated Hb level (≥13.0 g/dL) in the GDM group and the non-GDM group of the present study was not noticeable, which showed that both groups entered pregnancy with the same Hb status.

Additionally, there was no significant association found between Hb level in the second trimester and GDM risk in the present study. Women in this study were intensively given iron supplements and maternal nutrition advice as anemia preventive measures during antenatal care to avoid iron deficiency anemia (IDA), which is known to be related to adverse pregnancy outcomes (24, 49). However, there were no data on dietary intake in this study that can be used to examine the total iron intake on top of iron supplements. A study in a university hospital in Kuala Lumpur reported that Hb cut-off at 11.5 g/dL in the second trimester was significantly associated with GDM. The GDM group also had higher hematocrit levels than the non-GDM group, which was an indicator of high iron status (34). Chen et al. (50) also found a significant association between high iron status in the second trimester with GDM. Women with the highest quintile serum ferritin level (>131 pmol/L) had a twofold increase in GDM risk compared to women in the lower quintile. Likewise, a study in Iran reported a significant association between high serum iron in the second trimester with GDM occurrence. The association remained significant even after adjusting for confounders (51). These three studies, however, only screened GDM in 24–28 weeks of pregnancy and not during the first trimester (<14 weeks of gestation). Therefore, overt diabetes could have been included in the analysis which affected the significant findings. High iron status is hypothesized to affect glucose metabolism which increases diabetes risk, and perhaps women with overt diabetes may manifest the same high iron effects since pre-pregnancy (14, 52, 53). On the contrary, this study only included cases with true GDM that were confirmed during booking (<14 weeks of gestation).

Unchanged Hb status in the second trimester compared to booking was found to be significantly associated with GDM. A plausible explanation for the significant association between unchanged Hb and GDM is that unchanged Hb during pregnancy may reflect iron overload. The mean Hb for the unchanged subgroup at booking was 12.51 ± 1.10 g/dL which is similar to the cut-off point for high Hb concentration in the first trimester that increases GDM risk in several previous studies in middle-east and Asian countries (41–46). The anemia rate in the unchanged Hb subgroup was maintained at 9.4% in the second trimester compared to booking. The expansion of plasma volume in the unchanged Hb subgroup was less likely to have profound changes in Hb possibly because of iron supplementation that reduced the anemia incidence as pregnancy progresses (54, 55). Thus, the high Hb concentration > 12.5 g/dL at booking was maintained in the second trimester.

Most studies hypothesized that the significant association between elevated Hb and GDM risk was confounded by nutritional status, specifically iron, through diet or supplementation that contributed to iron overload (38–40, 43, 56). However, a woman could exhibit iron deficiency without anemia despite having a normal Hb (≥12.0 g/dL). Iron deficiency anemia (IDA) is a late manifestation of iron deficiency; thus, Hb value alone may not be the most sensitive indicator of body iron storage (24, 57–59). Even though Hb is one of the reliable tests for assessing iron status, it is more suitable to assess iron depletion and anemia (57, 59–61). As there is increasing evidence of a relationship between high iron status and increased risk of GDM, it is necessary to use other sensitive iron biomarkers, particularly serum ferritin, to know the iron status not only among anemia women.

Routine iron intake during early pregnancy <14 weeks until the second trimester in non-deprived iron women, could increase the risk of GDM (62). However, iron supplement intake in this study did not contribute to GDM risk. Women with unchanged Hb in this study could have also consumed high iron from food other than iron supplements which lead to iron accumulation. Another reason could be the elevated serum ferritin that is also known to be associated with the risk of GDM. Serum ferritin may be an indicator of iron stores as well as inflammation of cells (63, 64). Nevertheless, there was no information on dietary iron and serum ferritin status in this study to confirm the associations, other than iron supplementation.

### Strengths of the study

The strength of the study is that it was a multicentre study that included women from rural and urban areas. The sample size of the study was also large. Furthermore, the study included analyzes of Hb change impact on GDM risks which was an area with limited published Malaysian studies available. To avoid selection bias in the study, several strategies were undertaken. First, the inclusion criteria were restricted to healthy pregnant women. Second, disease conditions of the women that could affect the results of GDM were also specified. Third, compliance with antenatal care appointments was used as a sample selection to ensure the consistency of antenatal care data. Fourth, the study only included women who underwent two times OGTTs, which were done during the first and second trimester. This confirmed the glycemic status of the women during early pregnancy; thus, women with diabetes in pregnancy can be identified and excluded from the study. Finally, being a retrospective design, there was no recall bias involved as all the data were based on what had been recorded in the antenatal cards.

### Limitations of the study

This study is not without limitations. Incomplete records in the antenatal cards caused the loss of many subjects from contributing to the study. There is also a possibility of observational bias arising from documenting errors. The study only extracted data from the antenatal cards of HCs copy. The documentation by the health personnel was handwritten in the mother’s copy and HCs copy. Therefore, there is a high chance of wrongly copied data in either antenatal card. The present study only evaluates maternal Hb which does not reflect the actual iron status. The other iron indices to confirm the plasma expansion effects, such as serum iron, ferritin, transferrin, and total iron-binding capacity (TIBC) in this study, are limited as the blood investigations are only applicable to women with anemia during pregnancy. Moreover, the present study did not have data on the dietary iron intake that will give information on the total iron intake during pregnancy. The iron supplementation in the present study was based on the records of iron supplements prescribed with limited compliance records in the antenatal cards. Thus, reports on the iron supplement intake were based on the assumption that the women consumed the supplement as indicated. Therefore, the study is not presenting the actual iron supplementation of the women that could have implicated the no association findings between GDM and iron supplementation.

## Conclusion

The significant association between unchanged Hb status and GDM risk observed in the present study indicates the need to revise the policies on iron supplementation for pregnant women, especially for high Hb status during pre-pregnancy or non-deprived iron women. However, this suggestion does not repudiate the iron supplement prophylaxis dose of 30 mg daily for pregnant women recommended by the WHO, as Malaysian women of reproductive age are still prone to iron deficiency. Future studies that employ prospective design are warranted to evaluate the mechanisms involved in the unchanged Hb-GDM relationship to further understand the connection. Such findings could give a better insight into developing appropriate health and nutrition strategies to reduce the increasing rate of GDM in Malaysia.

## Data availability statement

The original contributions presented in the study are included in the article/supplementary files, further inquiries can be directed to the corresponding author.

## Ethics statement

The studies involving human participants were reviewed and approved by Medical Research and Ethics Committee National Institute of Health, Malaysia. The ethics committee waived the requirement of written informed consent for participation.

## Author contributions

HZS and MSZ conceived and designed the study and responsible for data management and analysis. HZS conducted the research including data collection and interpreted the data and wrote the first draft of the manuscript. MSZ supervised and critically revised the manuscript for important and intellectual content. AG supervised and revised the manuscript for important and intellectual content. All authors contributed to the article and approved the submitted version.

## Conflict of interest

The authors declare that the research was conducted in the absence of any commercial or financial relationships that could be construed as a potential conflict of interest.

## Publisher’s note

All claims expressed in this article are solely those of the authors and do not necessarily represent those of their affiliated organizations, or those of the publisher, the editors and the reviewers. Any product that may be evaluated in this article, or claim that may be made by its manufacturer, is not guaranteed or endorsed by the publisher.
